# Construction of layered hierarchical CoMoO_4_ nanostructured arrays for supercapacitors with enhanced areal capacitance

**DOI:** 10.1098/rsos.181592

**Published:** 2019-01-30

**Authors:** XiaoYan Hu, Hai Wang, SanMei Jin, Heng Wang

**Affiliations:** 1 School of New Energy and Electronic Engineering, Yancheng Teachers University, Yancheng 224007, People's Republic of China; 2 School of Mathematics and Physics, China University of Geosciences, Wuhan 430079, People's Republic of China

**Keywords:** CoMoO_4_ nanoplate array, layered hierarchical, hydrothermal, supercapacitor, areal capacitance

## Abstract

Layered hierarchical CoMoO_4_ nano-structured arrays grown on nickel foam were designed and synthesized by a two-step hydrothermal method following by annealing. With the increase in the nearly three times loading mass of active materials, the specific capacitance of the layered hierarchical CoMoO_4_ nano-structured arrays only shows a slight loss compared with the single-layer CoMoO_4_ nano-structured arrays, which dramatically improved the areal capacitance from 2.47 to 6.79 F cm^−2^. Also, the layered hierarchical CoMoO_4_ nano-structured arrays showed 94.8% capacitance retention after 2500 cycles, which is mainly due to the well-designed layered hierarchical structure and good conductivity.

## 1. Introduction

Supercapacitors, with longer lifespan, better safety and faster charge–discharge capability, are being considered as most promising energy-storage devices [1–5]. Among the supercapacitor materials, transition metal oxides, CoO*_X_*, NiO and MnO_2_, have been widely investigated because of their high theoretical capacitance [6–8]. In recent years, ternary metal oxides, CoMoO_4_, NiCo_2_O_4_ and MnMoO_4_, have evoked tremendous interest for their better electrical conductivity and higher redox activity compared to single component [9,10]. CoMoO_4_ is advantageous because of its low cost and non-toxic properties, and it also exhibits excellent electrochemical properties [11–13]. Wang and co-workers reported CoMoO_4_ nanoplates on Ni foam which shows a remarkable specific capacitance of 2526 F g^−1^ at 4 mA cm^−2^. However, the mass loading is about 0.5 mg cm^−2^ and relatively low, the areal capacitance is 1.26 F cm^−2^ [14]. In a practical application, high areal capacitance leads to higher energy density and output power supply, which is more significant. To further improve the CoMoO_4_-related supercapacitor device and areal capacitance, Liu and co-workers [15,16] synthesized hierarchical CoMoO_4_@NiMoO_4_ core–shell nanosheet arrays and CoMoO_4_@MnO_2_ core–shell structure on nickel foam, which had a high areal capacitance of 3.3 F cm^−2^ and 2.27 F cm^−2^, respectively.

As seen, core–shell structure could lead to an improved specific capacitance and electrochemical behaviours. However, the shell is usually very thin and the low mass loading still limits the practical application. When the thickness is too great, the shell would in turn restrain the ion transfer to the inner core. Herein, a novel layered hierarchical nano-structured CoMoO_4_ on the three-dimensional nickel foam is designed to get high areal capacitance. The first layer is designed as CoMoO_4_ nanoplate arrays and the second layer is designed as nanoflower-like structure deposited on the first layer. Such a layered hierarchical structure possesses the following advantages: (i) CoMoO_4_ nanoplate arrays on the nickel foam have a high electrochemical activity and abundant redox sites, and, as ternary metal oxide nanoarrays, CoMoO_4_ nanoplate arrays can provide direct charge transfer channel for the second layer; (ii) the second layer CoMoO_4_ nanoflower structure on the CoMoO_4_ nanoplate arrays is not closely packed and electrolyte could penetrate into the first layer directly; (iii) a high mass loading could be facilely achieved for this structure and the areal capacitance is improved. As a result, the designed layered hierarchical nano-structured CoMoO_4_ on the three-dimensional nickel foam exhibits a high areal capacitance of 6.79 F cm^−2^ at 5 mA cm^−2^, which is much higher than single-layer CoMoO_4_ nanoplate arrays. Also, at a high current density of 50 mA cm^−2^, the layered hierarchical nano-structures still have 94.8% capacitance retention after 2500 cycles, which implies that our structure design is efficient to improve the areal capacitance and can be applied to construct the structure of other electrode materials.

## 2. Experimental and computational section

**Figure 1.**
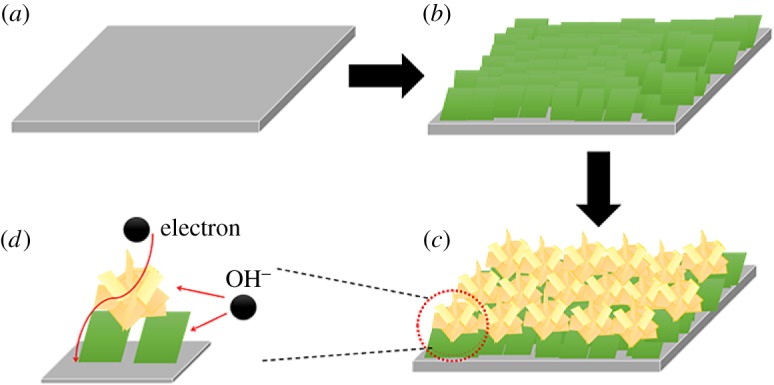
(*a*) Substrate nickel foam; (*b*) single-layer CoMoO_4_ nanoplate arrays on the nickel foam; (*c*) layered hierarchical CoMoO_4_ nanostructured arrays on the nickel foam; (*d*) electron and ion transfer in the layered hierarchical structure.

All the reagents were purchased from Sinopharm Chemical Reagent Co. Ltd; Co(NO_3_)_2_·6H_2_O, 98%; Na_2_MoO_4_·7H_2_O, 98%. The thickness of the nickel foam is about 1.6 mm and the porosity is 96.7%, 110 PPI. The brief fabrication procedure is illustrated in figure 1. The detail is given below.

### 2.1. Synthesis of single-layer CoMoO_4_ nanoplate arrays on nickel foam

The substrate nickel foam was rinsed with ethanol, deionized water and hydrochloric acid solution. To obtain the CoMoO_4_ nanoplate arrays on the nickel foam, 2.5 mmol Co(NO_3_)_2_·6H_2_O and 2.5 mmol NaMoO_4_·7H_2_O were mixed and stirred for 10 min, then the solution was transferred into a Teflon-lined stainless steel autoclave liner with the PTFE tape wrapped nickel foam immersed. The liner was sealed in a Teflon-lined stainless steel autoclave and maintained in an electric oven at 180°C for 12 h. After the reaction, the nickel foam with the arrays were rinsed with deionized water several times and dried at 60°C for 5 h. Finally, the sample was heated at 300°C for 2 h and single-layer CoMoO_4_ nanoplate arrays on the nickel foam was obtained. The weight of the active material was 1.85 mg cm^−2^. This structure is denoted as SL-CoMoO_4_.

### 2.2. Synthesis of layered hierarchical CoMoO_4_ nanostructured arrays on the nickel foam

To obtain the layered hierarchical nanostructured array on the nickel foam, the obtained single-layer CoMoO_4_ nanoplate arrays on the nickel foam were put into the same solution as described above. The same hydrothermal reaction was repeated at 180°C for 9 h. After the reaction, the nickel foam with the layered structures were rinsed with deionized water several times and dried at 60°C for 5 h. Finally, the sample was heated at 300°C for 2 h and layered hierarchical CoMoO_4_ nanostructured arrays on the nickel foam were obtained. The weight of the active material was 4.95 mg cm^−2^. This structure is denoted as LH-CoMoO_4_.

### 2.3. Characterization methods

The products were characterized using X-ray diffraction (XRD, PANalytical Empyrean, Cu Ka radiation; *λ* = 1.5418 Å) and field-emission scanning electron microscopy (FESEM, JEOL JSM-6700F, 10 kV); the mass of the electrode materials was measured on an AX/MX/UMX balance (METTLER TOLEDO, maximum = 5.1 g; *d* = 0.001 mg). To characterize the electrochemical behaviours, CHI 660D (CH Instruments Inc., Shanghai) electrochemical workstation was used in a three-electrode electrochemical cell using a 6 M KOH aqueous solution as electrolyte. Electrochemical impedance spectroscopy was tested by applying an AC voltage with 5 mV amplitude in a frequency range from 100 kHz to 0.1 Hz at open circuit potential. To test the electrochemical behaviours of LH-CoMoO_4_ electrodes, 2 × 0.5 cm^2^ nickel foam with the active material was cut as the working electrode, Ag/AgCl electrode was used as the reference electrode and Pt foil was used as the counter electrode. The electrolyte is 6 M KOH aqueous solution. SL-CoMoO_4_ was also tested for comparison.

## 3. Results and discussion

**Figure 2.**
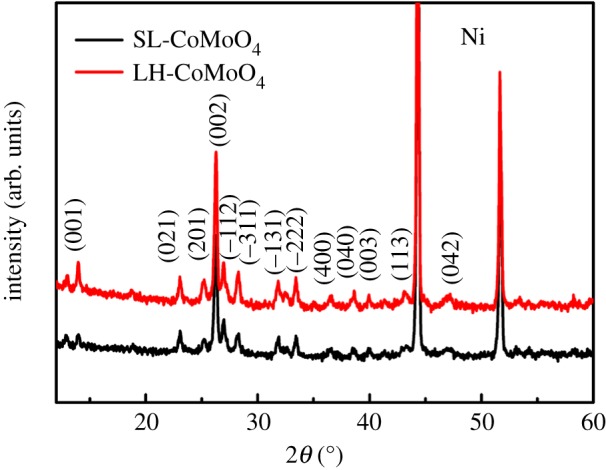
XRD patterns of the SL-CoMoO_4_ and LH-CoMoO_4_ on the nickel foam.

XRD patterns of the SL-CoMoO_4_ and the LH-CoMoO_4_ on the nickel foam are displayed in figure 2. As shown, for the single-layer CoMoO_4_, two ultrahigh peaks come from the substrate Ni, other peaks can be indexed to the (001), (021), (201), (002), (−112), (−311), (−131), (−222), (400), (040), (003), (113), (042) lattice planes, which corresponds to the monoclinic CoMoO_4_ (JCPDS card No. 21-0868). After second hydrothermal reaction for 9 h, XRD spectrum of LH-CoMoO_4_ shows no obvious difference from the SL-CoMoO_4_, which indicates product obtained during the second step is also CoMoO_4_.

**Figure 3.**
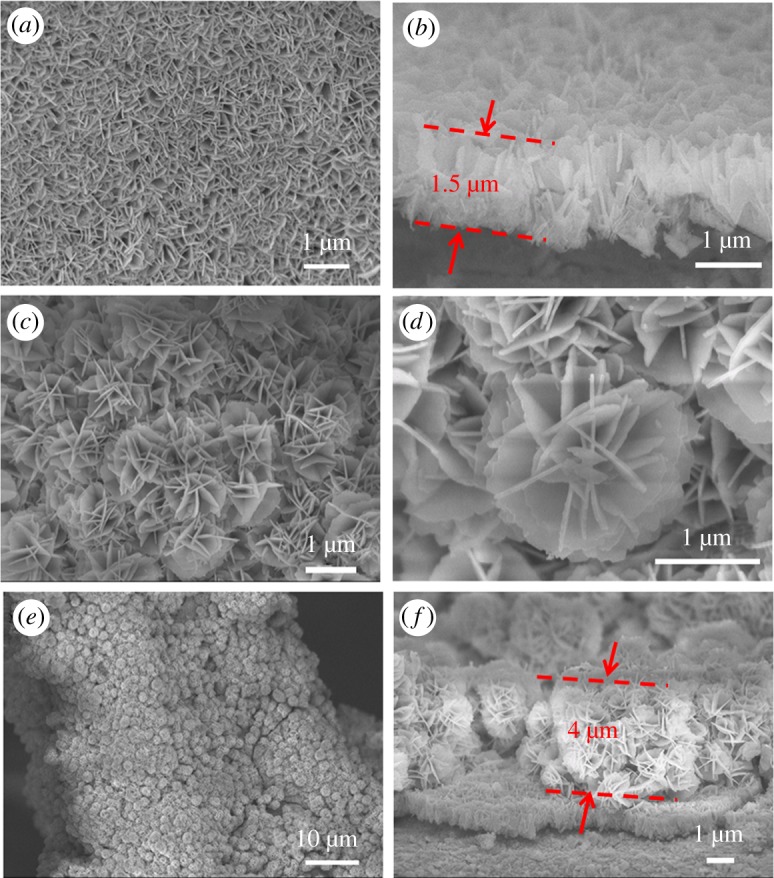
SEM images: (*a*) top view image of SL-CoMoO_4_; (*b*) cross-section image of the SL-CoMoO_4_; (*c*) top view image of LH-CoMoO_4_; (*d*) high magnification of single nanoflower from LH-CoMoO_4_; (e) low magnification of LH-CoMoO_4_; (*f*) cross-section image of LH-CoMoO_4_.

The scanning electron microscope (SEM) image of SL-CoMoO_4_ on the nickel foam is shown in figure 3*a,b*. Figure 3*a* displays the top view of SL-CoMoO_4_; obviously, the nanoplate arrays grew vertically and uniformly on the nickel foam. The cross-section of SL-CoMoO_4_ is shown in figure 3*b*. The thickness of the array is about 1.5 µm. Figure 3*c–f* is the SEM images of LH-CoMoO_4_. As shown in figure 3*c,d*, the second layer CoMoO_4_ is mostly constituted of CoMoO_4_ nanoflowers. The diameter of the CoMoO_4_ nanoflowers is about 1–2 µm and the nanoflower is also made up of randomly oriented CoMoO_4_ nanoplates. Figure 3*e* displays low magnification of LH-CoMoO_4_ on single nickel wire on the nickel foam, which indicates that the first layer nanoplate arrays are covered with CoMoO_4_ nanoflowers. Figure 3*f* displays the cross-section image of LH-CoMoO_4_ on the nickel foam. Obviously, the nanoflowers closely contact with each other and are directly deposited on the nanoplate array layer. The thickness is about 4 µm. It was worth noting that the CoMoO_4_ nanoflower-like structure did not completely cover the first layer because some gaps existed among the nanoflower structures. Thus, the electrolyte can penetrate into the first layer directly, which implies that our layered nanoflower structure would not restrain the ion transport on the basis of increased mass loading.

From the XRD, SEM and other related works, we can conclude the growth process of our LH-CoMoO_4_ as follows: at the first hydrothermal reaction stage, Co^2+^ and MoO_4_^2−^ ions combined and formed nanoparticles as the seeds on the three-dimensional nickel foam at a supersaturated solution. After that, the nanoparticle grew in a preferred crystal growth direction and formed vertically oriented nanoplate arrays. At the second hydrothermal reaction stage, because the first layer has grown on the substrate, there is no flat surface for the growth of the seed. The seed directly nucleated in the solution and grew up into nanoplate randomly, formed the nanoflowers [10,17]. Finally, the nanoflowers deposited on the first layer of CoMoO_4_ and LH-CoMoO_4_ was obtained.

**Figure 4.**
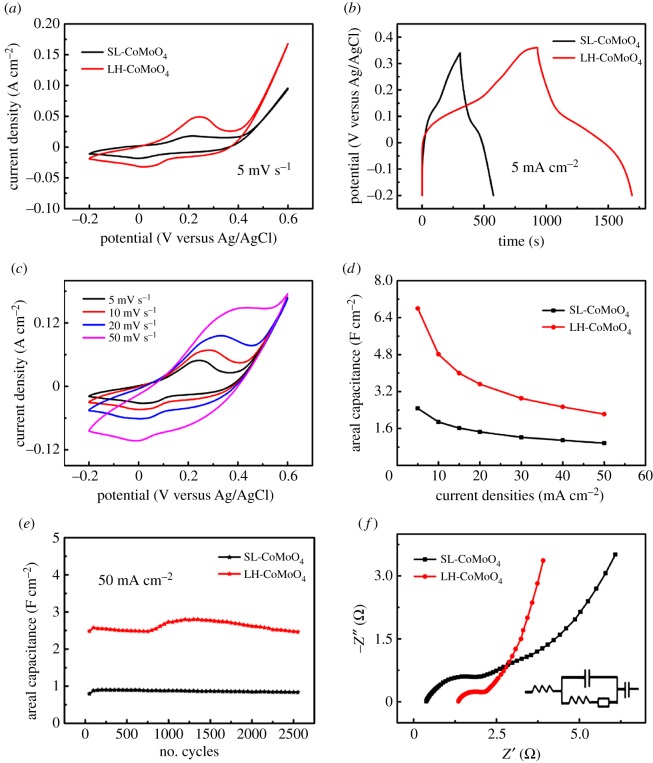
Electrochemical test of SL-CoMoO_4_ and LH-CoMoO_4_: (*a*) CV comparison at 5 mV s^−1^; (*b*) charge–discharge curves at 5 mA cm^−2^; (*c*) CV test of LH-CoMoO_4_ at different scanning rates; (*d*) areal capacitance at different current densities; (*e*) cycle performance at 50 mA cm^−2^; (*f*) Nyquist plots.

Figure 4*a* displays the cyclic voltammetry (CV) of SL-CoMoO_4_ and LH-CoMoO_4_ at 5 mV s^−1^. Redox peaks correspond to the redox reactions of Co^2+^/Co^3+^ and Co^3+^/Co^4+^. Obviously, the LH-CoMoO_4_ electrode has a higher CV loop area than SL-CoMoO_4_, which shows a better capacitive behaviour. At a current density of 5 mA cm^−2^, LH-CoMoO_4_ can discharge for 761.4 s, which is much higher than that of SL-CoMoO_4_, as shown in figure 4*b*. Figure 4*c* displays the CV test of LH-CoMoO_4_ at different scanning rates in the potential window −0.2–0.6 V. Areal capacitance of SL-CoMoO_4_ and LH-CoMoO_4_ at different current densities are shown in figure 4*d*. At a current density of 5 mA cm^−2^, LH-CoMoO_4_ exhibits high areal capacitance of 6.79 F cm^−2^, which is much higher than the SL-CoMoO_4_ of 2.47 F cm^−2^. It is also higher than other CoMoO_4_-related work (such as CoMoO_4_@NiMoO_4_ core–shell nanosheet arrays on nickel foam, 3.3 F cm^−2^ at 8 mA cm^−2^; CoMoO_4_@MnO_2_ core–shell structure aligned on Ni foam, 2.27 F cm^−2^ at 3 mA cm^−2^ [15,16]).

Meanwhile, the mass of the electrode materials loading on the nickel foam was measured on a balance. The loading mass was calculated to be 1.85 and 4.95 mg cm^−2^ for LH-CoMoO_4_ and SL-CoMoO_4_, respectively. Considering the loading mass, the specific capacitances of LH-CoMoO_4_ and SL-CoMoO_4_ are 1331.3 and 1372.2 F g^−1^, respectively. Although the mass loading has increased three times, specific capacitance only shows a slight loss. Even at a high rate of 50 mA cm^−2^, LH-CoMoO_4_ still remained areal capacitance of 2.22 F cm^−2^, showing good rate capability. The cycle tests of the SL-CoMoO_4_ and LH-CoMoO_4_ at 50 mA cm^−2^ are shown in figure 4*e*. Both SL-CoMoO_4_ and LH-CoMoO_4_ show excellent cycle performance. After 2500 cycles, LH-CoMoO_4_ still retains 94.8% capacitance of the initial capacitance. Especially deserving to be mentioned, after 1000 cycles, there even exists a rising cycling performance which can be ascribed to the layered hierarchical structure and the compact but porous nanostructure: (i) the bottom of the second layer is closely connected to the top of the first layer with the reaction progressing, so that the structure damage caused by volume expansion during the cycling process was alleviated, resulting in enhanced stability. (ii) The compact but porous nanostructure also helps to alleviate the structure damage caused by volume expansion during the cycle test. Figure 4*f* shows the Nyquist plots with the equivalent circuit in the inset of SL-CoMoO_4_ and LH-CoMoO_4_, respectively. The diameter of the semicircle in the mid-frequency is the charge-transfer resistance (*R*_ct_) of the electrode material. For SL-CoMoO_4_ and LH-CoMoO_4_, the *R*_ct_ values are 2.32 and 1.18 Ω, which indicates that the LH-CoMoO_4_ still had high charge conductivity. These results convince that the LH-CoMoO_4_ is a very promising supercapacitor electrode material and the layered hierarchical nanostructure is an efficient structure to improve the areal capacitance.

## 4. Conclusion

A layered hierarchical nano-structured CoMoO_4_ grown on nickel foam has been designed by a facile two-step hydrothermal reaction following annealing treatment. The LH-CoMoO_4_ shows a high areal capacitance of 6.79 F cm^−2^, which is much higher than the SL-CoMoO_4_ of 2.47 F cm^−2^. Meanwhile, the LH-CoMoO_4_ shows good conductivity and excellent cycle performance, which is mainly due to the well-designed nanostructure. Such outstanding electrochemical behaviours confirm that our structure design is efficient to improve the areal capacitance and can be applied to construct the structure of other electrode materials.

## Supplementary Material

Reviewer comments

## Data accessibility

There are no additional data to accompany this manuscript. All relevant datasets are within the main body of the manuscript. All protocols and software used are stated fully in the methodology section. There are no coding lists for this research.
